# Influence of sodium Bituminosulfonate and Doxycycline on signal molecules relevant for rosacea symptoms

**DOI:** 10.1038/s41598-025-02796-0

**Published:** 2025-05-23

**Authors:** Ann Sophie Rein, Marina Henke, Sophie Brünner, Sonja Luckhardt, Anna-Lena Zodel, Annette Sethmann, Susanne Schiffmann

**Affiliations:** 1 Fraunhofer Institute for Translational Medicine and Pharmacology (ITMP), Theodor-Stern-Kai 7, 60596 Frankfurt am Main, Germany; 2 Department of Clinical Pharmacology, Goethe-University Hospital Frankfurt, Theodor-Stern- Kai 7, 60590 Frankfurt/Main, Germany; 3 Ichthyol-Gesellschaft Cordes, Hermanni & Co. (GmbH & Co.) KG, Sportallee 85, 22335 Hamburg, Germany

**Keywords:** Sodium Bituminosulfonate, Doxycycline, Rosacea, INOS, VEGF, LL37, Molecular medicine, Preclinical research

## Abstract

Rosacea is treated among others by doxycycline and sodium bituminosulfonate dry substance (SBDS). We addressed the molecular mechanism(s) underlying the therapeutic benefit of SBDS and doxycycline. Therefore, we investigated whether SBDS or doxycycline regulates the expression of signal molecules relevant for inflammatory and angiogenic effects. Cyclooxygenase 1 (COX1) and COX2 activity assays were assessed in primary human monocytes. The release of nitric oxide (NO) and their synthesizing enzyme inducible nitric oxide synthase (iNOS), VEGF and LL37 release were determined in lung epithelial cells A549, mast cells HMC 1.2 or in normal human epidermal keratinocytes (NHEKs), respectively. The IC_50_ values of SBDS for the inhibition of recombinant human COX-1 and COX-2 were 1.9 and 8.3 µg/ml, respectively. In an inflammatory state (COX-2 expression) 100 µg/ml SBDS reduced PGE_2_ and TXB_2_ release in primary human monocytes. 25 µg/ml SBDS inhibited the mRNA and protein expression of iNOS. Moreover, 50 µg/ml SBDS and 30 µg/ml doxycycline inhibited the release of VEGF in an inflammatory state in HMC 1.2 cells. Both drugs did not affect LL37 release. Doxycycline did not affect intracellular NO levels and iNOS expression. SBDS and doxycycline mediate anti-inflammatory and anti-angiogenic effects through similar but also different signaling molecules.

**Supplementary Information:**

The online version contains supplementary material available at 10.1038/s41598-025-02796-0.

## Introduction

Rosacea is a prevalent, chronic facial inflammatory disorder that affects about 5% of the global population^1^. It is characterized by symptoms such as papules, pustules, telangiectasia, and temporary flushing or persistent erythema accompanied by burning and stinging sensations^2^. These symptoms often lead to social phobia and stress among patients^3,4^. Rosacea is believed to be influenced by various factors, including bacterial proteases, heat, stress/irritants, and UVB radiation^5^. These factors activate the immune system, leading to inflammation, pain, dilation of blood vessels, and the formation of new blood vessels in the skin. As a result, the clinical symptoms of rosacea are initiated and intensified.

The pathophysiology of rosacea remains to be elucidated, but numerous factors are known to contribute. Among these, a dysregulation of immune cells specifically macrophages and mast cells as well as tissue cells such as keratinocytes are clearly implicated^6^. In lesions of rosacea patients an increased level of cytokines (e.g., IL8, TNFα), cyclooxygenases (COX), vascular endothelial growth factor (VEGF), inducible nitric oxide synthase (iNOS) and cathelicidin as well as an increased number of immune cells and differentiated keratinocytes were detected^7–10^. It is hypothesized that macrophages activated by external stimuli leading to an increased release of eicosanoids, cytokines, chemokines, VEGF and thereby contributing to rosacea development^8,11^. Mast cells attracted by chemokines may release vascular endothelial growth factor (VEGF) and promoting thereby angiogenesis^12^. Neutrophils and keratinocytes activated by the pro-inflammatory environment release the anti-microbial peptide LL-37^13^. LL-37 was recently linked to the generation of reactive oxygen species (ROS)^14^. Nitric oxide (NO) product of iNOS can specifically induce intestinal mucosal inflammation and alter physiological processes in the skin, including vasodilation, inflammation, and immunomodulation, leading to the clinical manifestations of flushing and erythema associated with rosacea^15^. Moreover, ROS induce production of VEGF^16^, which together with IL-1β and TNF-α, contribute to the vascular hyperreactivity seen in rosacea^17^. In summary, a variety of factors that activate the immune system, induce connective tissue damage (e.g. ROS), vascularization (e.g. VEGF) or vasodilation (e.g. eicosanoids) drive the development of rosacea^18^.

The importance of these factors in the development of rosacea becomes evident from the modes of action of approved drugs for rosacea. Common treatments are doxycycline, topical azelaic acid and sodium bituminosulfonate dry substance (SBDS), among others. It was shown that topical azelaic acid inhibits expression of KLK5 and cathelicidins in keratinocytes and/or in treated rosacea patients^6^. SBDS inhibits in neutrophils the release of LL37, VEGF, elastase, ROS and inhibits the activity of KLK5, 5-LO and MMP9^19^. Doxycycline inhibits the production and activity of MMP9, inhibits the activity of KLK5, and reduces pro-inflammatory cytokine release and the synthesis of ROS^6^.

In rosacea, patients experience redness of the skin due to inflammation, angiogenesis, and vasodilation^6^. These symptoms can be treated with SBDS and doxycycline^20,21^. SBDS is an non-biological complex drug (NBCD) derived from sulphur-rich oil shale with anti-inflammatory properties^19^. A comprehensive two-dimensional gas chromatography coupled to an electron ionization high-resolution time-of-flight mass spectrometer (GC × GC-HR-ToF–MS) analysis revealed the structural class of the thiophenes as the most abundant in SBDS^22^. In 1953, the formulation SBDS was introduced onto market, and is approved in Germany and indicated for the treatment of rosacea.

The clinical effectiveness of tetracyclines such as doxycycline in the treatment of rosacea has been mainly attributed to their anti-inflammatory action, inhibitory effects on angiogenesis, leukocytic chemotaxis, inflammatory cytokines, and matrix metalloproteinase^23^. Doxycycline (40, 100 and 200 mg/day) is approved for the treatment of rosacea^23^. 100 mg doxycycline administrated once lead to a plasma c_max_ of about 1.5 µg/ml^24^.

A direct comparison of doxycycline and SBDS regarding its effect on signal molecules in in vitro experiments is missing. Moreover, some of the signaling molecules are derived from more than one cell type for example LL37 is released by keratinocytes and neutrophils, while VEGF is released by mast cells and neutrophils^25,26^. To understand how SBDS and doxycycline can mediate anti-angiogenic and anti-inflammatory effects, we conducted experiments on mast cells, keratinocytes, epithelial cells, and macrophages. We analyzed various signal molecules like NO, PGE_2_, TXB_2_ and proteins like iNOS, COX-1, COX-2, VEGF, LL37 involved in the synthesis or breakdown of anti-angiogenic and anti-inflammatory signaling molecules.

## Results

### SBDS inhibited COX-1 and COX-2 activity

**Fig. 1:**
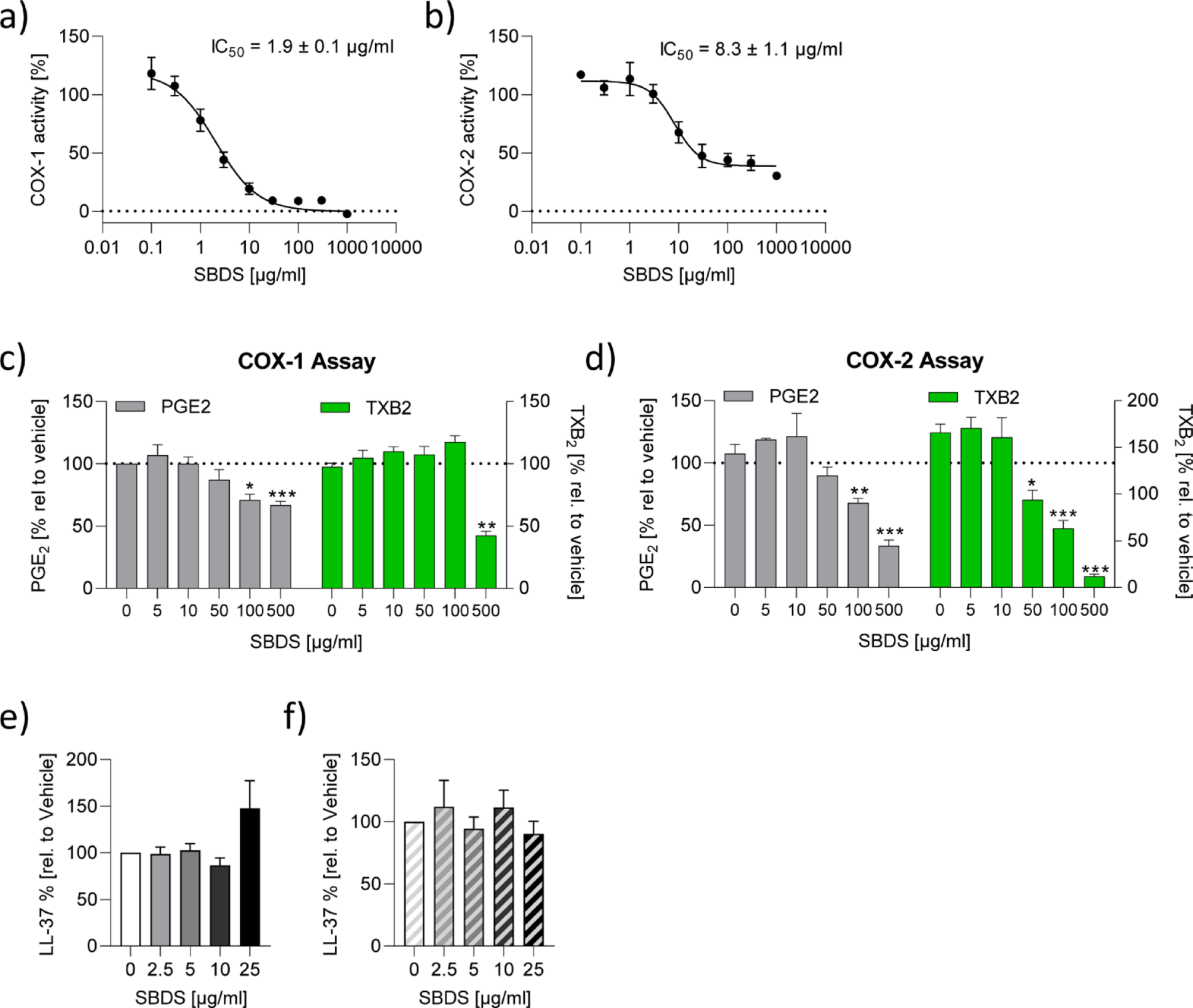
SBDS inhibited COX-1 and COX-2 and did not affect LL-37. (**a**/**b**) SBDS in a concentration range from 0.1–1000 µg/ml were tested with a COX-1 (**a**) or COX-2 (**b**) activity assay. The experiments were performed in three replicates. The IC_50_ values were calculated using a non-linear fitting model (GraphPad Prism Software 9.2.0). (**c**) For the cellular COX-1 assay, primary human CD14^+^ monocytes were treated with SBDS or vehicle for 60 min. To start COX-1 reaction its substrate arachidonic acid was added. **(d**) For the cellular COX-2 assay, primary human CD14^+^ monocytes were pretreated with ASA to block COX-1 activity and COX-2 expression was induced by LPS treatment for 16 h. Subsequently monocytes were treated with SBDS or vehicle for 30 min. To start COX-2 reaction its substrate arachidonic acid was added. The products PGE_2_ and TXB_2_ were determined by ELISA. To obtain fold induction the PGE_2_ and TXB_2_ levels of samples treated with SBDS were related to control samples. The experiment was achieved with monocytes from three different donors. (**e**/**f**) NHEKs were preincubated for 3 h with SBDS at the indicated concentrations and stimulated with 2.5 µg/ml LPS (**f**; striped bars) or left untreated (e; blank bars) for 6 h. The release of LL-37 was determined by ELISA. The experiments were performed in three replicates. To calculate statistical significance Mixed-effects analysis with Dunnett`s multiple comparisons test was used. * *p* < 0.05, ** *p* < 0.01 and *** *p* < 0.001 indicate statistical significance between treated samples and vehicle samples. Abb. COX, cyclooxygenase; PGE_2_, prostaglandin E2; TXB_2_, thromboxane 2.

Ammonium bituminosulfonate (Ichthyol^®^) inhibits the prostaglandin endoperoxide synthase isolated from sheep vesicular gland with an IC_50_ value of 300 µg/ml^27^. However Schewe et al. did not specify whether COX1 and/or COX2 was investigated. Therefore, we tested whether SBDS can affect the activity of human cyclooxygenase (COX), an enzyme responsible for synthesizing the precursor lipid PGH_2_ which is metabolized by PGE synthase or thromboxane synthase to PGE_2_ and TXB_2_, respectively. We performed an activity assay using recombinant human COX-1 and COX-2. The IC_50_ values for COX-1 and COX-2 were determined to be 1.9 ± 0.1 µg/ml and 8.3 ± 1.1 µg/ml, respectively (Fig. 1a/b). SBDS completely inhibited COX-1, whereas COX-2 activity was inhibited to about 70%.

To investigate if the inhibition of COX-1 by SBDS can also be observed in a cellular set-up, primary human monocytes which constitutively express COX-1 were used. The enzyme was activated by the addition of its substrate arachidonic acid. The human primary monocytes were treated with increasing concentrations of SBDS and levels of PGE_2_ and TXB_2_ were measured. The positive control acetylsalicylic acid (ASA) (20 µg/ml) inhibited COX-1 using PGE_2_ as read-out to 73% and using TXB_2_ as read-out to 92%. 1 µM SC-650 inhibited COX-1 using PGE_2_ or TXB_2_ as read-out to 52% and 76%, respectively (Supplemental Fig. 1a). Interestingly, 100 µg/ml of SBDS inhibited the release of PGE_2_, while 500 µg/ml was required to inhibit TXB_2_. However, the reduction in PGE_2_ was only about 30%, whereas TXB_2_ was reduced by about 60% (Fig. 1c).

For the cellular COX-2 assay, primary human monocytes were stimulated with LPS (lipopolysaccharide) to induce COX-2 expression and pretreated with ASA to inhibit constitutively expressed COX-1. COX-2 was activated by the addition of its substrate arachidonic acid. As expected, the COX-1 inhibitor SC-560 (5 µM) did not inhibit COX-2 using PGE_2_ or TXB_2_ as read-out. The positive control NS-398 (5 µM) inhibited COX-2 using PGE_2_ as read-out to 63% and using TXB_2_ as read-out to 69%. 5 µM diclofenac inhibited COX-2 using PGE_2_ or TXB_2_ as read-out to 82% and 92%, respectively (Supplemental Fig. 1b). Interestingly, at a concentration of 100 µg/ml, SBDS reduced the level of PGE_2_, and 50 µg/ml of SBDS reduced the level of TXB_2_. 500 µg/ml SBDS reduced PGE_2_ levels by about 70% and TXB_2_ levels by about 90% (Fig. 1d). These findings suggest that SBDS has a stronger inhibitory effect on the release of PGE_2_ and TXB_2_ in an inflammatory (COX-2) environment compared to a normal (COX-1) environment within a cellular system.

### SBDS did not affect LL-37 release in NHEKs

Innate immune activation leads to upregulation of keratinocyte-derived toll-like receptor 2 (TLR2) promoting the expression of the antimicrobial peptide cathelicidin, which is subsequently activated to bioactive LL-37 by kallikrein 5 (KLK-5) protease, leading to erythema and angiogenesis^28^. Recently, we demonstrated that SBDS inhibited KLK5 the enzyme that metabolizes cathelicidin to LL-37 with an IC_50_ value of 7.6 µg/ml^19^. Moreover, we observed that SBDS reduced the release of LL-37 in neutrophils. Besides neutrophiles also keratinocytes are main producers of LL-37^5^, therefore we investigated if SBDS also inhibits the release of LL-37 in keratinocytes. We used non-toxic concentrations of SBDS (0–20 µg/ml), identified by a viability assay (Supplemental Fig. 2a). Normal human epidermal keratinocytes (NHEKs) were pretreated with SBDS and stimulated with the TLR2 agonist LPS or left unstimulated. Astonishingly, SBDS had no influence on LL-37 release in NHEKs independent of the activation status (Fig. 1e/f). These data indicate that the effect of SBDS on the inhibition of LL-37 release is cell type dependent.

### SBDS inhibited iNOS expression in A549 cells

**Fig. 2:**
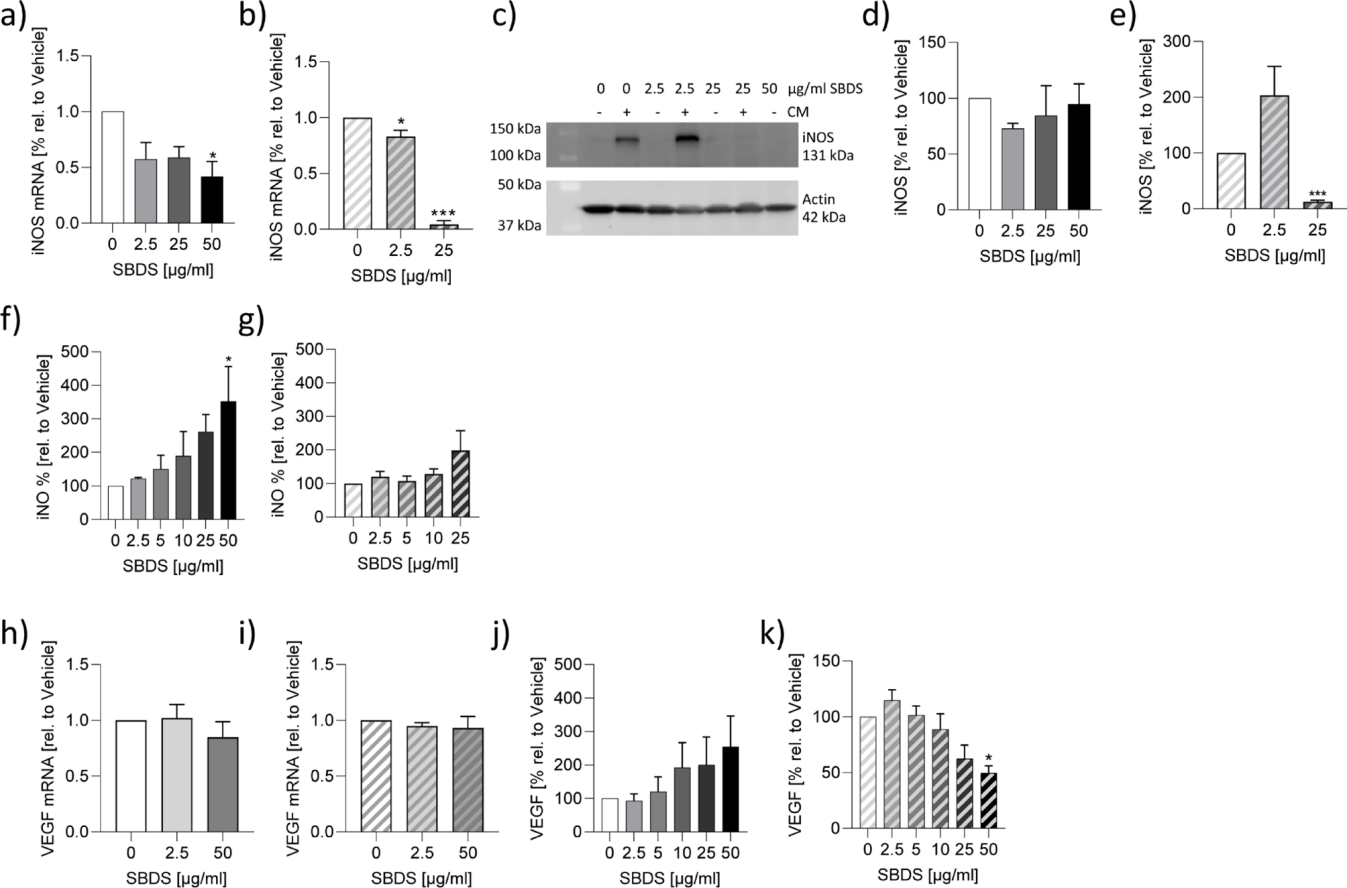
SBDS did inhibit iNOS expression and VEGF release. (**a**-**g**) The iNOS mRNA (**a**/**b**), protein (**c**-**e**) expression and iNO levels (**f**/**g**) were determined in A549 cells stimulated (striped bars) with 5 ng/ml IL1β, 5 ng/ml IFNγ and 5 ng/ml TNFα or unstimulated (blank bars) in presence or absence of SBDS in the indicated concentrations. (**a**/**b**) iNOS and GADPH mRNA expression was determined by qPCR. iNOS levels were normalized to GAPDH. SBDS treated samples were related to control samples. (**c**) iNOS and actin levels were determined by western blot technique. (**d**/**e**) The optical densitometry analysis was achieved with Image Lab 6.0 software. The iNOS levels were normalized to actin. SBDS treated samples were related to control samples. (**f**/**g**) iNO levels were determined by staining of NO with 5 µM DAF-FM-DA and analysing by flow cytometry. The MFI of SBDS-treated samples were related to control samples. (**h**-**k**) The VEGF mRNA (**h**/**i**) and protein (**j**/**k**) expression was determined in HMC 1.2 cells stimulated (striped bars) with 25 ng/ml PMA and 200 nM calcium ionophore A23187 or unstimulated (blank bars) in presence or absence of SBDS in the indicated concentrations. (**h**/**i**) VEGF and GADPH mRNA expression were determined by qPCR. VEGF levels were normalized to GAPDH. SBDS treated samples were related to control samples. (**j**/**k**) VEGF levels collected from the supernatant were determined by ELISA. The VEGF level of SBDS treated samples were related to control samples. The experiments were repeated three - five times. To calculate statistical significance two-way ANOVA with Dunnett`s multiple comparisons test was used. *p < 0.05, ** *p* < 0.01 and *** *p* < 0.001 indicate statistical significance between treated samples and vehicle samples. Abb. CM, cytokine mixture; DOX, doxycycline; iNOS, inducible nitric oxide synthase; iNO, intracellular nitric oxide; VEGF, vascular endothelial growth factor.

Since SBDS inhibited the release of LL-37 an inducer of reactive oxygen species in some cell types, we investigated whether SBDS also affects the NO synthesis. We used non-cytotoxic concentrations of SBDS, identified by a viability assay (Supplemental Fig. 3a). We observed an IC_50_ value of 9 µg/ml for NO inhibition in murine RAW 264.7 macrophages (Supplemental Fig. 3b). To translate this effect into the human system we used A549 lung epithelial cells, since there is no known stimuli to induce NO release ex vivo in human macrophages^29^. Non-cytotoxic concentrations identified by a viability assay were applied (Supplemental Fig. 2b). We investigated whether SBDS could alter iNOS expression by treating A549 cells with various concentrations of SBDS. 50 µg/ml SBDS inhibited the basal iNOS mRNA expression (Fig. 2a), whereas 2.5 µg/ml SBDS significantly inhibited cytokine mix-induced iNOS mRNA expression (Fig. 2b). Interestingly, also the cytokine mix-induced iNOS protein expression was significantly reduced by 25 µg/ml, whereas the basal iNOS level was not influenced by SBDS (Fig. 2c-e). Astonishingly, the inhibition of iNOS did not translate into a reduction of intracellular NO levels in cytokine mix stimulated A549 cells (Fig. 2f/g).

### SBDS inhibited VEGF release

Next, we investigated the effect of SBDS on VEGF release and the expression of VEGF mRNA in mast cells, since in rosace mast cells release VEGF and contribute thereby to angiogenesis^30^. Recently, it was suggested that mast cells which release VEGF play a role in connecting innate immunity, nerves, and blood vessels in the rosacea development. Moreover, an increased number of mast cells were observed in rosacea lesions^30^. Therefore, we examined whether SBDS may affect the VEGF release in the mast cell line HMC 1.2. Non-cytotoxic SBDS concentrations were used (Supplemental Fig. 2c). HMC 1.2 cells were treated with SBDS and stimulated with phorbol-12-myristate-13-acetate (PMA) and calcium ionophore to produce/release VEGF. SBDS did not induce the VEGF mRNA expression (Fig. 2h), nor did inhibit the PMA/calcium ionophore-induced VEGF mRNA expression in HMC 1.2 cells (Fig. 2i). Mast cells store VEGF intracellularly, which is then released upon stimulation^31^. SBDS did not affect the release of VEGF in unstimulated cells (Fig. 2j), whereas 50 µg/ml SBDS inhibited the PMA/Ca ionophore-induced VEGF release (Fig. 2k). These data indicate that SBDS prevents the exocytosis of VEGF stored in vesicles in an inflamed state.

### Doxycycline inhibited VEGF release

**Fig. 3:**
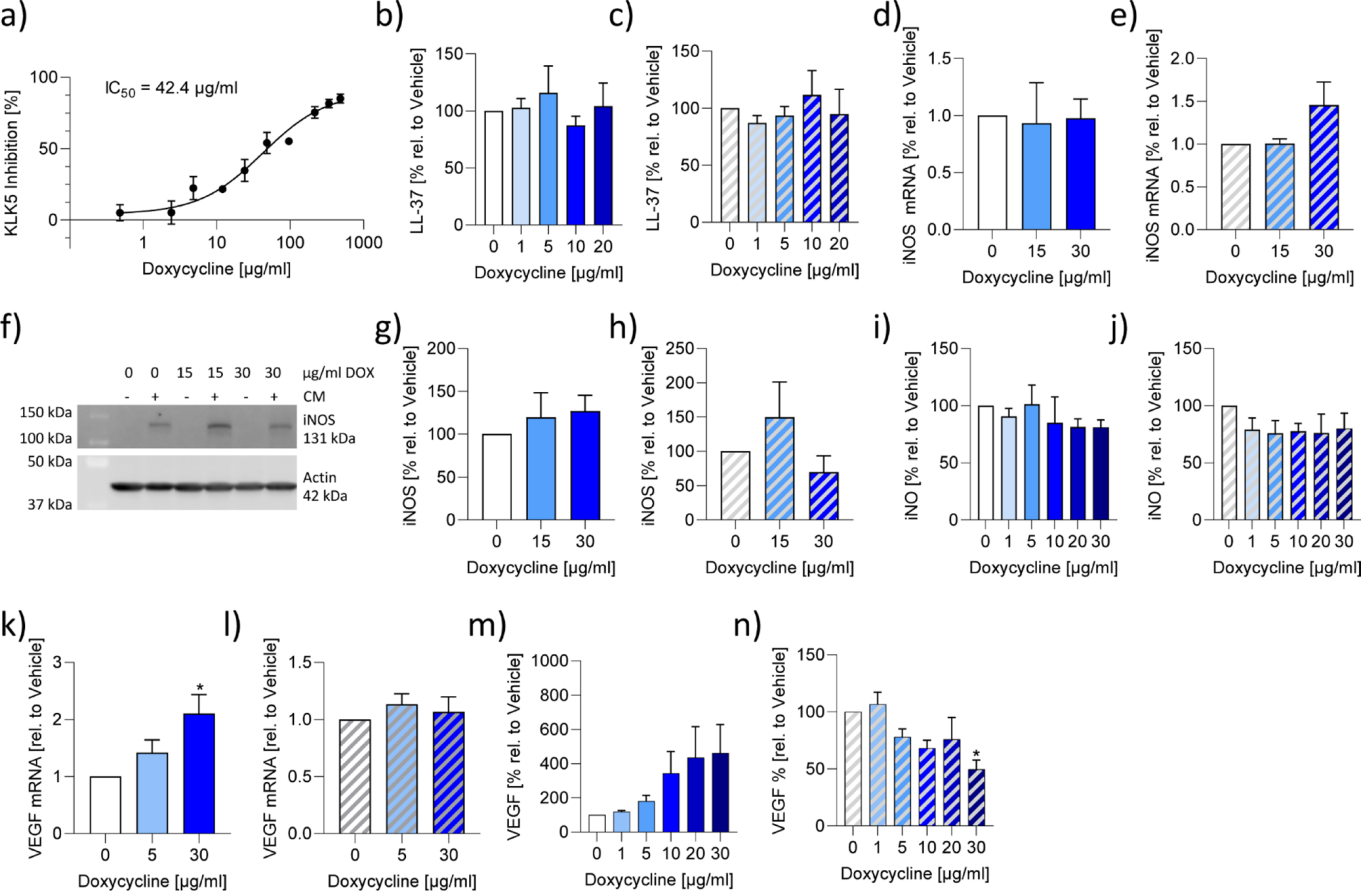
Effects of doxycycline on KLK5, LL37, iNOS, iNO and VEGF. (**a**) Human recombinant KLK5 was preincubated with doxycycline for 5 min and incubated with its fluorogenic substrates for 5 min and the product was detected spectrophotometrically. The IC_50_ values was calculated using a non-linear fitting model (GraphPad Prism Software 9.2.0). (**b**/**c**) NHEKs were preincubated for 3 h with doxycycline at the indicated concentrations and stimulated with 2.5 µg/ml LPS (c; striped bars) or left untreated (b; blank bars) for 6 h. The release of LL-37 was determined by ELISA. (**d**-**j**) The iNOS mRNA (**d**/**e**), protein (**f**-**h**) expression and iNO levels (**i**/**j**) were determined in A549 cells stimulated (striped bars) with 5 ng/ml IL1β, 5 ng/ml IFNγ and 5 ng/ml TNFα or unstimulated (blank bars) in presence or absence of doxycycline in the indicated concentrations. (**d**/**e**) iNOS and GADPH mRNA expression was determined by qPCR. iNOS levels were normalized to GAPDH. Doxycycline treated samples were related to control samples. (**e**-**h**) iNOS and actin levels were determined by western blot technique. (**g**/**h**) The optical densitometry analysis was achieved with Image Lab 6.0 software. The iNOS levels were normalized to actin. Doxycycline treated samples were related to control samples. (**i**/**j**) iNO levels were determined by staining of NO with 5 µM DAF-FM-DA and analysing by flow cytometry. The MFI of doxycycline treated samples were related to control samples. (**k**-**n**) The VEGF mRNA (**k**/**l**) and protein (**m**/**n**) expression was determined in HMC 1.2 cells stimulated (striped bars) with 25 ng/ml PMA and 200 nM calcium ionophore A23187 or unstimulated (blank bars) in presence or absence of doxycycline in the indicated concentrations. (**k**/**l**) VEGF and GADPH mRNA expression were determined by qPCR. VEGF levels were normalized to GAPDH. Doxycycline treated samples were related to control samples. (**m**/**n**) VEGF levels collected from the supernatant were determined by ELISA. The VEGF level of doxycycline treated samples were related to control samples. The experiments were repeated three – five times. To calculate statistical significance two-way ANOVA with Dunnett`s multiple comparisons test was used. *p < 0.05 indicate statistical significance between treated samples and vehicle samples. Abb. CM, cytokine mixture; DOX, doxycycline; iNOS, inducible nitric oxide synthase; iNO, intracellular nitric oxide; VEGF, vascular endothelial growth factor.

To compare the mode of actions of SBDS and doxycycline, we also investigated the impact of doxycycline on various signaling molecules. We previously determined an IC_50_ value of 7.6 µg/ml for SBDS in inhibiting KLK5^19^. In contrast, doxycycline revealed an IC_50_ value of 42.4 µg/ml (Fig. 3a). Non-cytotoxic doxycycline concentrations were used for the cellular assays (Supplemental Fig. 2a-c). Doxycycline did not affect the basal and LPS-induced release of LL-37 in NHEKs (Fig. 3b/c). Interestingly, doxycycline did not influence intracellular NO levels, iNOS protein and iNOS mRNA expression in both basal and inflammatory state in A549 cells (Fig. 3d-j). 30 µg/ml doxycycline induced the synthesis of VEGF mRNA in unstimulated HMC 1.2 cells (Fig. 3k), but did not influence PMA and calcium ionophore stimulated VEGF mRNA expression (Fig. 3l). However, 30 µg/ml doxycycline inhibited VEGF release induced by PMA and calcium ionophore in HMC 1.2 cells (Fig. 3n). These findings indicate that doxycycline inhibits VEGF signaling in the inflammatory state.

### Combination therapy of Doxycycline and SBDS had no beneficial effect on signal molecules

**Fig. 4:**
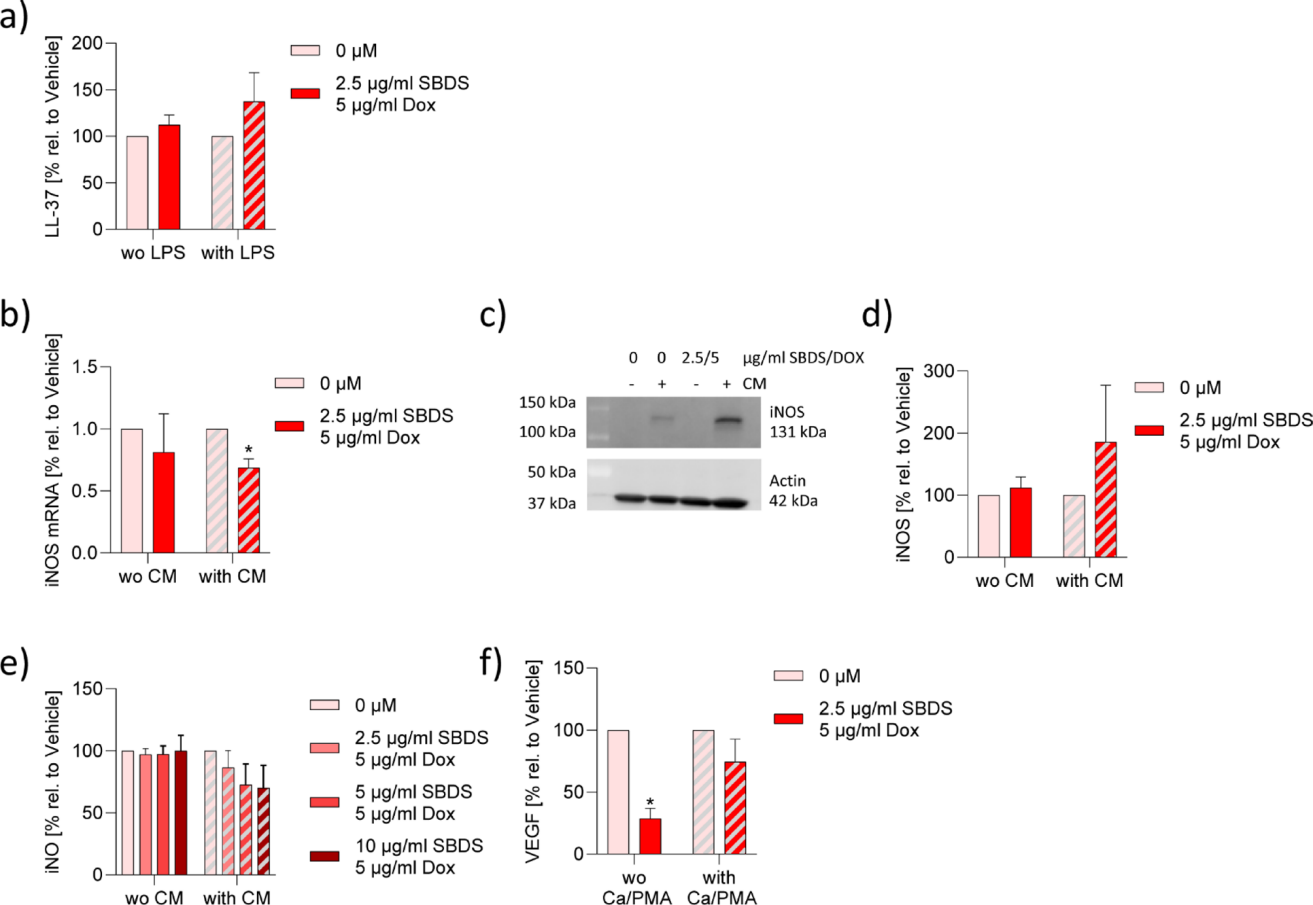
Combination treatment of SBDS and doxycycline has no effect on LL37, iNOS, iNO and VEGF. (**a**) NHEKs were preincubated with 2.5 µg/ml SBDS and 5 µg/ml doxycycline for 3 h and stimulated with 2.5 µg/ml LPS (striped bars) or left untreated (blank bars) for 6 h. The release of LL-37 was determined by ELISA. b-d) The iNOS mRNA (**b**) and protein (c/d) expression was determined in A549 cells stimulated (striped bars) with 5 ng/ml IL1β, 5 ng/ml IFNγ and 5 ng/ml TNFα or unstimulated (blank bars) in presence or absence of 2.5 µg/ml SBDS and 5 µg/ml doxycycline. (**b**) iNOS and GADPH mRNA expression was determined by qPCR. iNOS levels were normalized to GAPDH. SBDS and doxycycline treated samples were related to control samples. (**c**) iNOS and actin levels were determined by western blot technique. (**d**)The optical densitometry analysis was achieved with Image Lab 6.0 software. The iNOS levels were normalized to actin. SBDS and doxycycline treated samples were related to control samples. (**e**) iNO levels were determined by staining of NO with 5 µM DAF-FM-DA and analysing by flow cytometry. The MFI of doxycycline and SBDS treated samples were related to control samples. (**f**) The VEGF protein expression was determined in HMC 1.2 cells stimulated (striped bars) with 25 ng/ml PMA and 200 nM calcium ionophore A23187 or unstimulated (blank bars) in presence or absence of 2.5 µg/ml SBDS and 5 µg/ml doxycycline. VEGF levels collected in the supernatant were determined by ELISA. The VEGF level of SBDS and doxycycline treated samples were related to control samples. The experiments were performed in biological triplicates. To calculate statistical significance two-way ANOVA with Dunnett`s multiple comparisons test was used. *p < 0.05 indicate statistical significance between treated samples and vehicle samples. Abb. Ca, calcium ionophore; CM, cytokine mixture; DOX, doxycycline; iNOS, inducible nitric oxide synthase; iNO, intracellular nitric oxide; LPS, lipopolysaccharide; PMA, phorbol-12-myristate-13-acetate; VEGF, vascular endothelial growth factor.

Since combination treatments such as oral doxycycline (40 mg once/day) and azelaic acid 15% gel are also effective, safe, and well-tolerated in rosacea patients^20^, we investigated whether combining doxycycline and SBDS has any beneficial in vitro effects. We used non-effective concentrations of SBDS (2.5 µg/ml) and doxycycline (5 µg/ml) to investigate if the compounds have synergistic effects. The combination therapy had no effect on the release of LL37 (Fig. 4a), did not affect iNOS mRNA in the basal state and protein expression (Fig. 4b-d) as well as the iNO levels in the basal and inflammatory state (Fig. 4e). But it reduced the iNOS mRNA level in the inflammatory state and the basal level of VEGF (Fig. 4b/4f). These findings suggest that the combination therapy does not have huge beneficial effects on the investigated signaling molecules.

## Discussion

SBDS reduced the release of inflammatory mediators (PGE_2_ and TXB_2_) and the synthesis of iNOS. SBDS and doxycycline reduced the release of the growth factor VEGF in an inflammatory state. Our data confirmed the anti-inflammatory potential of SBDS and doxycycline by the use of various signal molecules released by relevant cell types. An aim of the study was to compare the effects and the molecular mechanism of SBDS and doxycycline. In Table 1 literature data and our results of both agents on several signal molecules are summarized.

**Table 1:** Summary of the effects of SBDS and Doxycycline on various proteins and signaling molecules.

Target enzyme	Assay type	SBDS	Doxycycline
KLK5	Activity	IC_50_: 8 µg/ml^19^	IC_50_: 42 µg/ml
5-LO	Activity	IC_50_: 33 µg/ml^19^	-
MMP9	Activity	IC_50_: 51 µg/ml^19^	Inhibition at 50 µg/ml^55^
COX-1	Activity	IC_50_: 1.9 µg/ml	-
COX-2	Activity	IC_50_: 8.3 µg/ml	-
MMP9	fMLP-stimulated Neutrophils	No effect^19^	-
	RANKL-stimulated RAW 264.7	-	Inhibition at 2 µg/ml^56^
LL37	LTB_4_-stimulated Neutrophils	Inhibition at 50 µg/ml^19^	-
	NHEKs	No effect	No effect
VEGF	PMA-stimulated Neutrophils	Inhibition at 50 µg/ml^19^	-
	PMA/Ca ionophor stimulated HMC 1.2 mast cells	Inhibition at 50 µg/ml	Inhibition at 30 µg/ml
ROS	PMA-stimulated Neutrophils	Inhibition at 50 µg/ml^19^	-
Ca^2+^	fMLP-stimulated Neutrophils	Inhibition at 50 µg/ml^19^	-
iNOS	Cytokin-mix stimulated A549	Inhibition at 25 µg/ml	No effect
iNO	Cytokin-mix stimulated A549	No effect	No effect
eNO	LPS-stimulated RAW 264.7	IC_50_: 9.5 µg/ml	IC_50_: 17.5 µg/ml Inhibition at 20 µg/ml^57^
PGE_2_ (COX-1)	Monocytes	Inhibition at 100 µg/ml	-
TXB_2_ (COX-1)	Monocytes	Inhibition at 500 µg/ml	-
PGE_2_ (COX-2)	LPS-stimulated Monocytes	Inhibition at 100 µg/ml	-
	LPS-stimulated RAW 264.7	-	Induction at 20 µg/ml^57^
TXB_2_ (COX-2)	LPS-stimulated Monocytes	Inhibition at 50 µg/ml	-

- Not determined.

**Fig. 5:**
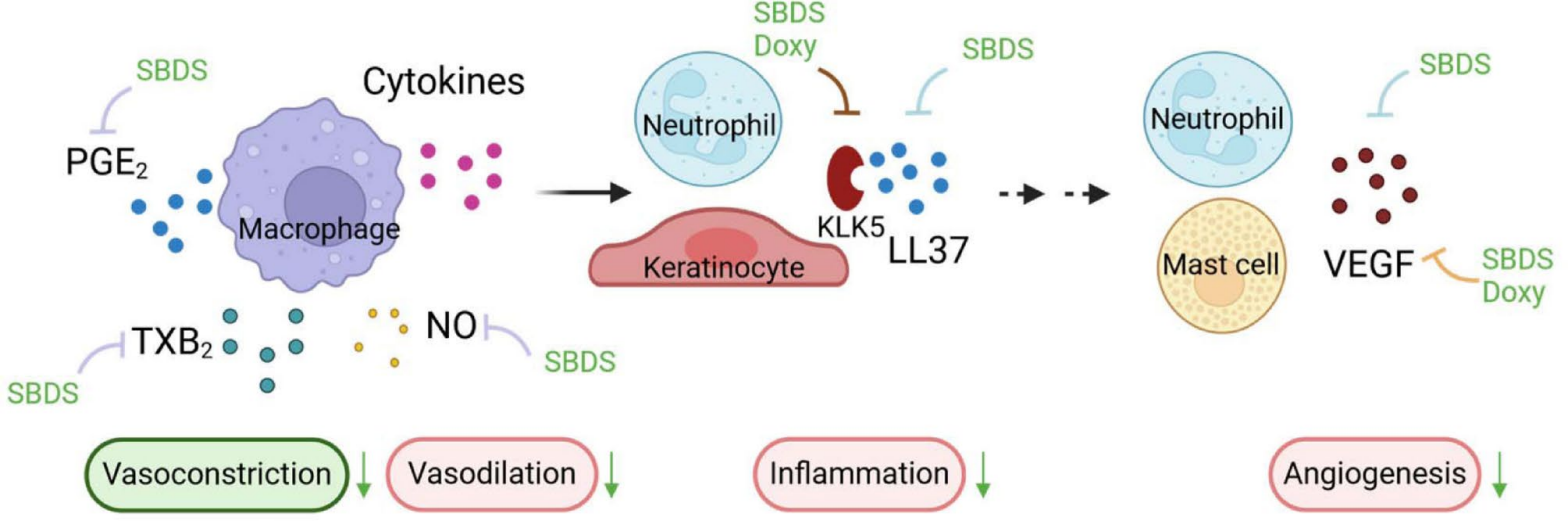
Potential contribution of SBDS and doxycycline in inflammatory and vascular-modifying pathways (created with Biorender). Macrophages release the vasoconstrictive TXB_2_. the vasodilatory NO and PGE_2_, which mediates either vasodilatory or vasoconstrictive effects depending on the interacting EP receptor. SBDS impairs the release of NO in murine macrophages and the release of PGE_2_ and TXB_2_ in human primary macrophages. Cytokines released by macrophages activate neutrophils and keratinocytes, which among others, release the antimicrobial peptide LL-37. KLK5 is responsible for cleaving the inactive precursor protein hCAP18 to form the active antimicrobial peptide LL-37. Both SBDS and doxycycline inhibit KLK5. SBDS specifically inhibits the release of LL-37 in neutrophils. LL-37 promotes the release of VEGF from neutrophils and mast cells. SBDS reduced the release of VEGF from both neutrophils and mast cells, while doxycycline reduced the release of VEGF from mast cells. Inhibitory arrows for macrophages are depicted in light purple, for neutrophils in blue, for the enzyme KLK5 in red, and for mast cells in yellow.

SBDS and doxycycline treatment of rosacea patients led to an improvement of symptoms potentially by reducing inflammation and vasodilation. Signal molecules that are responsible for vasodilation or vasoconstriction among others are NO, PGE_2_ and TXA_2_ (Fig. 5). NO mediates flow-mediated vasodilation, opposes vasoconstrictor effects, counteracts vascular stiffness and lowers blood pressure^32^. PGE_2_ can mediate either vasodilatory or vasoconstrictive effects depending on the receptor it interacts with. When PGE_2_ interacts with EP1 and EP3, it induces vasoconstriction, while it promotes vasodilatation when binding to EP2 and EP4 receptors^33^. TXA_2_ stimulates TXA_2_ receptor to induce vasoconstriction^34,35^. Interestingly, SBDS inhibited in murine macrophages NO release and in human lung epithelial cells iNOS expression in an inflammatory state, but had no effect on NO synthesis in human lung epithelial cells. Moreover, SBDS inhibited the vasoconstrictor TXB_2_ (stable metabolite of TXA_2_) and the vasodilator PGE_2_ in human monocytes. Since SBDS led to a reduction of redness in rosacea patients, these data indicate that the effect of SBDS on NO and TXB_2_ in vitro are potentially not relevant for the in vivo observed effect. The inhibition of PGE_2_ can potentially contribute to the clinical effects of SBDS, but for a final conclusion the expression levels of the EP receptors in rosacea would be needed. Furthermore, the beneficial effects of SBDS on the reduction of redness in rosacea patients may also stem from its impact on inflammatory molecules produced by other cell types present in the skin and/or lesions, such as neutrophils^36^ Previous studies have already demonstrated that SBDS inhibits VEGF, LL-37, ROS, and Ca^2+^ release in neutrophils^19^.

We observed a decrease in iNOS expression for SBDS; however, this does not correspond to a reduction in iNO levels. Several factors may explain this observation: (1) The dye DAF-FM-DA used is not specific for NO and may react with other cellular molecules or directly with a component of SBDS, as it is known to react with thiols^37^ One structural class of SBDS identified by two-dimensional gas chromatography coupled to an electron ionization high-resolution time-of-flight mass spectrometer consists of benzenethiols^22^ (2) SBDS may react with intracellular molecules to indirectly generate NO. (3) SBDS may influence the cellular redox state, thereby stabilizing the bioactivity of existing NO. Hoyt et al. observed for 30 µg/ml doxycycline an inhibition of NO release in murine lung epithelial cells^38^. In human lung epithelial cells, doxycycline did not affect NO synthesis. However, in rosacea patients treated with doxycycline a reduction of iNOS in the inflammatory infiltrate was observed^39^. It was not investigated whether TXB_2_ release is influenced by doxycycline, however Attur et al. showed that doxycycline induced an increase of COX-2 expression and PGE_2_ release in LPS-treated macrophages indicating that potentially also TXB_2_ a metabolite of PGH_2_ (product of COX-2 synthesis and precursor of TXB_2_) is increased. Since the production of PGH_2_ by COX-2 is the rate limiting step in prostaglandin synthesis, an increase in COX-2 is linked to an increase of the 5 majors bioactive prostaglandins (PGE_2_, PGI_2_, PGD_2_, PGF_2_, and TXA_2_) through their respective tissue-specific synthases, when they are expressed^40^. In macrophages thromboxane synthase is expressed (https://www.proteinatlas.org) and therefore a doxycycline induced increase of COX-2 may be linked to an increase in TXB_2_. In summary, SBDS and doxycycline potentially mediate their vasoconstrictive effects via different signal molecules.

We observed varying inhibitory effects of SBDS in the cellular system compared to the activity assay with recombinant protein. In the cellular assay, SBDS appears to have a greater impact on COX-2 than on COX-1, which contradicts the results from the activity assay. We hypothesize that this discrepancy may be due to the availability and metabolism of SBDS. However, the differing effects of SBDS on intracellular versus recombinant COX-2 and COX-1 can only be explained by these factors if different components of SBDS are responsible for inhibiting COX-1 and COX-2. This implies that the component inhibiting COX-1 has low permeability, while the component inhibiting COX-2 has high permeability.

In rosacea patients treated with doxycycline a reduction of cathelicidin LL37 was detected^39^. SBDS (IC_50_ = 8 µg/ml) and doxycycline (IC_50_ = 42 µg/ml) inhibited the activity of KLK5, the enzyme that processes the cathelicidin precursor protein (hCAP)18 to LL37^19^. This may translate to an inhibition of LL37 release in neutrophils by 50 µg/ml SBDS^19^, whereas in NHEKs no inhibition of LL37 was observed (Fig. 5). Also, doxycycline up to 30 µg/ml did not reduce the release of LL37 in NHEKs. However, Kanada et al. evaluated the ability of doxycycline to inhibit processing of cathelicidin by the addition of full-length recombinant cathelicidin hCAP18 to NHEKs and found that 225 µM doxycycline inhibits the release of LL37^41^. These data indicate that the inhibition of LL37 by SBDS and doxycycline seems to be cell type or concentration dependent. The inhibition of LL37 could be a mechanism how both drugs improve the clinical features of rosacea such as erythema, telangiectasia and inflammation, since in a mouse model a LL37 injection led to such clinical features possibly through the enhanced expression of IL1, IL6 and MMP9 in mast cells^42,43^. Additionally, LL-37 increases cytokine and chemokine liberation from leukocytes and has chemotactic effects on a large number of immune cells^44^.

In an inflammatory state, both doxycycline and SBDS inhibited the release of VEGF from mast cells, but have no effect on VEGF mRNA synthesis (Table 1; Fig. 5). VEGF is stored in vesicle in various cell types such as human neutrophils, human basophils, murine macrophages and human ovarian carcinoma cell lines CABAI and A2780^45–48^. Our findings in mast cells confirm previous results obtained in neutrophils that SBDS prevents the release of primary and secondary granules and also demonstrate this effect in mast cells^19^. In a basal state, SBDS and doxycycline seems to increase VEGF in mast cells (Figs. 2j and 3m). Interestingly, 10 µg/ml doxycycline has been shown to upregulate VEGF mRNA in murine cardiomyocytes and reduce it in murine endothelial cells, with no change in protein levels^49^. The VEGF mRNA upregulation in cardiomyocytes may contribute to the cardioprotective effects of doxycycline after myocardial infarction in rats and the reduced adverse post-myocardial infarction left ventricular remodelling in patients^50,51^. On the other hand, doxycycline has been shown to inhibit angiogenesis in endothelial cells, which aligns with the decreased VEGF mRNA expression observed in endothelial cells^49,52^. Moreover, in rosacea patients treated with doxycycline a reduction of VEGF was observed^39^. The potential effect of increased VEGF mRNA in mast cells needs to be further studied. In summary, the effect of doxycycline appears to be dependent on the cell type.

SBDS and doxycycline appear to exert its anti-inflammatory, anti-angiogenic and vasodilating effects in rosacea via different mechanisms and potentially target different cell types. Both inhibit the release of VEGF. Additionally, SBDS blocks the eicosanoid signaling. Our data suggest that SBDS possibly regulates anti-inflammatory and anti-angiogenic processes by suppressing PGE_2_ and the release of VEGF, respectively. Doxycycline may mediate its anti-angiogenic effect by suppressing VEGF. In summary, SBDS and doxycycline may have VEGF as target in common.

## Materials and methods

### Cells and reagents

A549 cells were obtained from Cell Bank of the JCRB (Japanese Collection of Research Bioresources)/HSRRB (Human Science Research Resources Bank) and cultured in DMEM F-12 medium supplemented with 10% FCS and 1% penicillin/streptomycin. HMC1.2 were purchased from Merck KGaA (SCC062, Darmstadt, Germany) and cultured in Iscove`s modified Dulbecco`s medium (IMDM) supplemented with 1.2 mM α-thioglycerol, 10% FCS and 1% penicillin/streptomycin. NHEK were purchased from PromoCell (C-12005, Heidelberg, Germany) and cultured in Keratinocyte Growth Medium 2 supplemented with 0.06 mM CaCl_2_ and 12.3 mM supplemental mix. RAW264.7 macrophages were a gift from Prof. Grösch (Goethe University Frankfurt) and cultured in RPMI 1640 medium supplemented with 10% FCS and 1% penicillin/streptomycin. Primary human monocytes were cultured in RPMI1640 medium supplemented with 10% FCS and 1% penicillin/streptomycin. All cells were cultured at 37 °C in a 5% CO_2_ atmosphere. Doxycycline was dissolved in water and further diluted in media. SBDS was dissolved in water or DMSO (COX-1/COX-2 assay) and further diluted in media. Oil shale-derived SBDS was provided by the Ichthyol-Gesellschaft. Orangu™ was purchased from Hiss Diagnostics GmbH (Freiburg, Germany). COX-1, COX-2 assay and TXB_2_ ELISA were obtained from Cayman Chemical Company (Ann Arbor, USA). PGE_2_ ELISA were purchased from Enzo Life Sciences GmbH (Lörrach, Germany).

### COX-1 and COX-2 activity assay

The assay was performed as described by the supplier. The COX-1 and COX-2 assay measures PGF_2α_ by SnCl_2_ reduction of PGH_2_ produced in the COX reaction. PGF_2α_ was quantified via a competitive ELISA. Increasing concentrations of SBDS (0–1000 µg/ml) were incubated with the recombinant human COX-1 or COX-2 for 10 min. Reaction was started by the addition of arachidonic acid, after 2 min SnCl2 was added to reduce PGH_2_ to PGF_2α_. PG-acetylcholinesterase conjugate was held constant while the concentration of the produced PGF_2α_ varied. PGF_2α_ and PG-acetylcholinesterase conjugate competed with the binding to PG antiserum. An increasing PGF_2α_ concentration led to a reduced binding of PG-acetylcholinesterase conjugate. PG-acetylcholinesterase conjugate and PGF_2α_ bound to PG antiserum, which was captured by a mouse monoclonal anti-rabbit antibody that had been attached to the well. After washing of the plate, the substrate (Ellman`s reagent) of the acetylcholinesterase was added and the product of this reaction was measured at 412 nm. The intensity of the absorbance was proportional to the amount of PG-acetylcholinesterase conjugate, which was inversely proportional to the amount of PG produced by COX-1.

### Cellular COX-1 and COX-2 assay

The COX-1 assay was performed as described by Demasi et al.^53^. Briefly, monocytes were isolated from buffy coat by positive selection using CD14^+^ microbeads (Miltenyi Biotec, Bergisch Gladbach, Germany) as described previously^54^. 4 × 10^5^ monocytes were pretreated with 10 µg/ml acetylsalicylic acid (ASA) in 200 µl RPMI medium (10% FCS), with SBDS (0, 5, 10, 50, 100, 500 µg/ml), 1 µM SC-560 or with vehicle (DMSO, Water) in 200 µl RPMI medium (10% FCS) for 30 min at 37 °C. The cells were washed once with 200 µl RPMI 10%FCS and once with 200 µl RPMI w/o FCS. 100 µM arachidonic acid (dissolved in EtOH and buffered with KOH) in 240 µl RPMI medium (without FCS) were added for 15 min at 37 °C. The supernatants were stored at −80 °C and the PGE_2_ and TXB_2_ level were determined by ELISA as described by the supplier.

The COX-2 assay was performed as described by Demasi et al.^53^. Briefly, 1 × 10^6^ monocytes were pretreated with 10 µg/ml ASA in 200 µl RPMI medium (10% FCS) for 30 min at 37 °C. The cells were washed twice with 200 µl RPMI medium (10% FCS). 10 µg/ml LPS in 200 µl RPMI medium (10% FCS) was added and incubated for 16 h at 37 °C to induce COX-2 expression. The cells were washed twice with 200 µl RPMI medium (without FCS). Cells were treated with SBDS (0, 5, 10, 50, 100, 500 µg/ml), 5 µM NS-398, 5 µM SC-560, 5 µM Diclofenac or with vehicle (DMSO, Water) in 200 µl RPMI medium (without FCS) for 30 min at 37 °C. The cells were washed twice with 200 µl RPMI medium (without FCS). 100 µM arachidonic acid (dissolved in EtOH and buffered with KOH) in 240 µl RPMI medium (without FCS) were added for 15 min at 37 °C. The supernatants were stored at −80 °C and PGE_2_ and TXB_2_ level were determined by ELISA as described by the supplier.

### LL-37 detection assay

40.000 NHEKs were seeded in 96 well plates and preincubated with 0, 2.5, 5, 10, 25 µg/ml of SBDS, 0, 1, 5, 10, 20 µg/ml doxycycline or vehicle for 3 h at 37 °C/5% CO_2_ atmosphere. 2.5 µg/ml LPS was added or the cells were left untreated and incubated for 6 h at 37 °C/5% CO_2_ atmosphere. The supernatant was collected and LL-37 was determined by ELISA, as recommended by the supplier (Hycultec GmbH, Beutelsbach, Germany). For analysis, the absorbance values from standard and from samples were corrected with that of the samples without cells. The concentration of LL-37 was extrapolated using the standard curve.

### iNOS mRNA detection

0.25 × 10^6^ A549 cells were pretreated with SBDS (0, 2.5, 25, 50 µg/ml) or doxycycline (0, 15, 30 µg/ml) in FCS-free DMEM/F 12 medium for 3 h and stimulated with a cytokine mixture consisting of 5 ng/ml IL1β, 5 ng/ml IFNγ und 5 ng/ml TNFα or left unstimulated for 18 h at 37 °C/5% CO_2_ atmosphere. Cells were washed, harvested and mRNA (RNAeasy Kit from Qiagen, Hilden, Germany) was isolated. 1 µg of mRNA were transcribed in cDNA by First Strand cDNA synthesis kit (Thermo Fisher Scientific, Waltham, USA) as recommended by the supplier. iNOS and GAPDH mRNA were determined by qPCR with EvaGreen^®^ Master Mix (Bio&Sell, Feucht/Nürnberg, Germany). iNOS forward primer: 5`GTGCTCTTTGCCTGTATGC; iNOS reverse primer: 5`CAGCTCAGCCTGTACTTATCC; GAPDH forward primer: 5`GCACCACCAACTGCTTAG; GAPDH reverse primer: 5`CCATCACGCCACAGTTTC. The data were analyzed by 2^−ΔΔCT^ method a relative quantification strategy for quantitative real-time polymerase chain reaction (qPCR) data analysis. The Bio-Rad CFX Manager 3.1 software was used. The mRNA expression levels were normalized to the reference gene glycerinaldehyde-3-phosphate-dehydrogenase (GAPDH) and related to the control samples.

### iNOS protein detection

1.75 × 10^6^ A549 cells were pretreated with SBDS (0, 2.5, 25, 50 µg/ml) or doxycycline (0, 15, 30 µg/ml) in FCS-free DMEM/F 12 medium for 3 h and stimulated with a cytokine mixture consisting of 5 ng/ml IL1β, 5 ng/ml IFNγ und 5 ng/ml TNFα or left unstimulated for 24 h at 37 °C/5% CO_2_ atmosphere. Cells were washed, harvested and lysed with RIPA buffer (ChemCruz/Santa Cruz Biotechnologie, Inc., Dallas, USA) supplemented with protease inhibitors (2 mM phenylmethanesulfonyl fluoride (PMSF), 1 mM Natrium orthovanadate und protease inhibitor cocktail). Protein concentration was determined by bicinchoninic acid. 100 µg protein were separated by SDS page and blotted. The membrane was blocked with 5% milk powder suspended in TBS-T buffer (20 mM Tris, 150 mM sodium chloride, 0.05% Tween 20) (at room temperature), stained with an anti-iNOS (1:500; 20609 S, Cell Signaling, Danvers, USA) for 2 days (at 4 °C) and mouse anti-actin (1:1000; CL594-66009, Proteintech Group Inc., Rosemont, USA) antibody for 2 h at room temperature. As second antibody goat anti-rabbit IgG-Alex Flour™ 488 (1:10000; A11008, Invitrogen/Thermo Fisher Scientific/Waltham, USA) were applied for 1 h at room temperature. The images of the blots were acquired with ChemiDocTM MP (Bio Rad Laboratories, Hercules, USA). Optical densitometry was determined by the Image Lab 6.0 software. The adjusted volume intensity of iNOS were related to actin. The relative iNOS levels from treated samples were related to vehicle treated samples.

### Intracellular NO level

1 × 10^4^ A549 cells were pretreated with SBDS (0, 2.5, 5, 10, 25, 50 µg/ml) or doxycycline (0, 1, 5, 10, 20, 30 µg/ml) in FCS-free DMEM/F 12 medium for 3 h and stimulated with a cytokine mixture consisting of 5 ng/ml IL1β, 5 ng/ml IFNγ und 5 ng/ml TNFα or left unstimulated for 24 h. Intracellular NO (iNO) was stained with 5 µM DAF-FM-DA for 30 min at 37 °C. Cells were washed three times with 100 µl Dulbecco´s Phosphate Buffered Saline and analysed by flow cytometry. MFI values of SBDS or doxycycline treated values with DAF-FM-DA were corrected with MFI values from cells treated with the substances without DAF-FM-DA. The corrected MFI values of SBDS or doxycycline treated cells were related to the corrected MFI values of vehicle-treated samples to obtain the relative iNO value.

### VEGF assay

25.000 or 500.000 HMC 1.2 cells were seeded on a 96-well plate (protein analysis) or 6-well plate (mRNA analysis), respectively, and treated with SBDS (0, 2.5, 5, 10, 25 µg/ml), doxycycline (0, 1, 5, 10, 20 µg/ml) or vehicle in medium with 5% FCS for 3 h at 37 °C/5% CO_2_ atmosphere. To investigate whether SBDS can prevent VEGF release/synthesis, HMC 1.2 mast cells were stimulated with 25 ng/ml PMA and 200 nM calcium ionophore A23187 for 6 h (mRNA) or 20 h (protein). To investigate whether SBDS induces VEGF release/synthesis, cells were left unstimulated for 6 h (mRNA) or 20 h (protein) at 37 °C/5% CO_2_ atmosphere.

Cells from the 6-well plate were centrifuged (300 g, 5 min, RT) and harvested for mRNA analysis. mRNA (RNAeasy Kit from Qiagen, Hilden, Germany) was isolated and 1 µg of mRNA were transcribed in cDNA by First Strand cDNA synthesis kit (Thermo Fisher Scientific, Waltham, USA) as recommended by the supplier. VEGF and GAPDH mRNA were determined by qPCR with EvaGreen^®^ Master Mix (Bio&Sell, Feucht/Nürnberg, Germany). VEGF forward primer: 5` GGGCAGAATCATCACGAAG; VEGF reverse primer: 5` CTCGATCTCATCAGGGTACTC; GAPDH forward primer: 5`GCACCACCAACTGCTTAG; GAPDH reverse primer: 5`CCATCACGCCACAGTTTC. The data were analyzed by 2^-ΔΔCt method using the Bio-Rad CFX Manager 3.1 software. The mRNA expression levels were normalized to the reference gene glycerinaldehyde-3-phosphate-dehydrogenase (GAPDH) and related to the control samples.

Supernatant from the 96-well plate was collected and the concentration of VEGF determined by ELISA (Thermo Fisher Scientific, Germany), as recommended by the supplier. For analysis, the absorbance values from standard and from test samples were corrected with the blank (standard: assay buffer; samples: medium with test substances), since the values of the controls decreased with increasing concentration of test substances. The concentration of VEGF was extrapolated using the standard curve.

### KLK5 assay

The KLK5 assay was performed as previously described^19^. Briefly, recombinant humane KLK5 enzyme (R&D Systems, Wiesbaden, Germany) and the fluorogenic substrate Boc-V-P-R-AMC substrate (Boc: t-Butyloxycarbonyl; AMC: 7-Amino-4-methylcoumarin) (R&D Systems, Wiesbaden, Germany) were used. rhKLK5 was diluted in 1 M NaH_2_PO_4_ pH 8 and doxycycline (0.48 µg/ml – 480 µg/ml) or water (background control (C) with enzyme without compound) was added and incubated for 5 min at RT. 100 µM Boc-V-P-R-AMC substrate was added and incubated for 5 min at RT. The fluorescence was detected with the Multimode Plate Reader (Tecan, Crailsheim, Germany) (Ex380 nm/Em460 nm). The inhibition was calculated with the following equation: (1 - (A - B1)/(C - B2)) * 100. A = RFU Test sample; B1 = basal RFU without enzymes with compound; B2 = basal RFU without enzymes with vehicle; C = RFU vehicle control with enzymes. The test samples (A) were corrected for the RFU of samples without enzyme but with test substance (B1), due to the high background signal of the test substance that was concentration dependent.

### Statistical analyses

Results are presented as means ± standard errors. The data were analysed with one-way ANOVA and or two-way ANOVA and with Dunnett’s multiple comparisons test. For all calculations and creation of graphs, GraphPad Prism 8 was used and *p* < 0.05 was considered the threshold for significance.

## Electronic supplementary material

Below is the link to the electronic supplementary material.

Supplementary Material 1

## Data Availability

Data will be made available from the corresponding author upon reasonable request.

## Abbreviations

ANOVA: Analysis of variance
ASA: Acetylsalicylic acid
COX: Cyclooxygenase
DAF-FM DA: 4-Amino-5-Methylamino-2',7'-Difluorofluorescein Diacetate
eNO: Extracellular nitric oxide
EP: Prostaglandin E receptor
FCS: Fetal calf serum
fMLP: Formyl-Methionyl-Leucyl-Phenylalanine
GAPDH: Glycerinaldehyd-3-phosphat-dehydrogenase
IL: Interleukin
iNO: Intracellular nitric oxide
iNOS: Inducible nitric oxide synthase
KLK5: Kallikrein 5
5-LO: 5 Lipoxygenase
LTB4: Leukotriene B4
LPS: Lipopolysaccharide
MMP9: Matrix metalloproteinase 9
NHEK: Normal human epidermal keratinocyte
NO: Nitric oxide
PBS: Phosphate-buffered saline
PGE_2_: Prostaglandin E2
PMA: Phorbol-12-myristate-13-acetate
ROS: Reactive oxygen species
SBDS: Sodium bituminosulfonate dry substance
TXB2: Thromboxane B2
TNFα: Tumor necrosis factor alpha
VEGF: Vascular endothelial growth factor
