# Supplementary material for: Influence of sodium Bituminosulfonate and Doxycycline on signal molecules relevant for rosacea symptoms
Source: Sci Rep. 2025 May 23;15:17894. doi: 10.1038/s41598-025-02796-0 (PMC12102399; doi:10.1038/s41598-025-02796-0)
Supplement: Supplementary file 1 — Supplementary Material 1 [file 41598_2025_2796_MOESM1_ESM.pptx]

## Slide 1
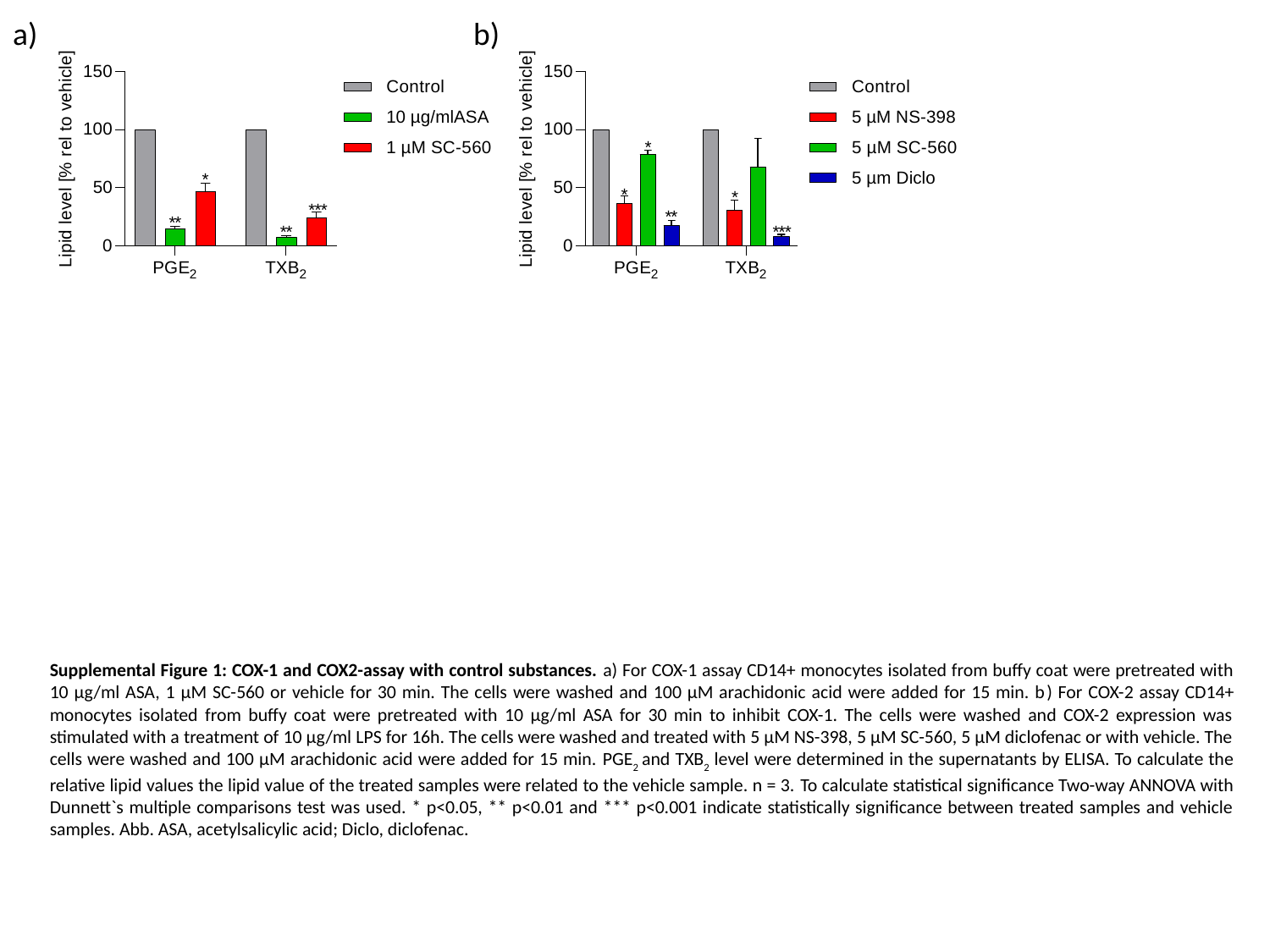

a)
b)
Supplemental Figure 1: COX-1 and COX2-assay with control substances. a) For COX-1 assay CD14+ monocytes isolated from buffy coat were pretreated with 10 µg/ml ASA, 1 µM SC-560 or vehicle for 30 min. The cells were washed and 100 µM arachidonic acid were added for 15 min. b) For COX-2 assay CD14+ monocytes isolated from buffy coat were pretreated with 10 µg/ml ASA for 30 min to inhibit COX-1. The cells were washed and COX-2 expression was stimulated with a treatment of 10 µg/ml LPS for 16h. The cells were washed and treated with 5 µM NS-398, 5 µM SC-560, 5 µM diclofenac or with vehicle. The cells were washed and 100 µM arachidonic acid were added for 15 min. PGE2 and TXB2 level were determined in the supernatants by ELISA. To calculate the relative lipid values the lipid value of the treated samples were related to the vehicle sample. n = 3. To calculate statistical significance Two-way ANNOVA with Dunnett`s multiple comparisons test was used. * p<0.05, ** p<0.01 and *** p<0.001 indicate statistically significance between treated samples and vehicle samples. Abb. ASA, acetylsalicylic acid; Diclo, diclofenac.

## Slide 2
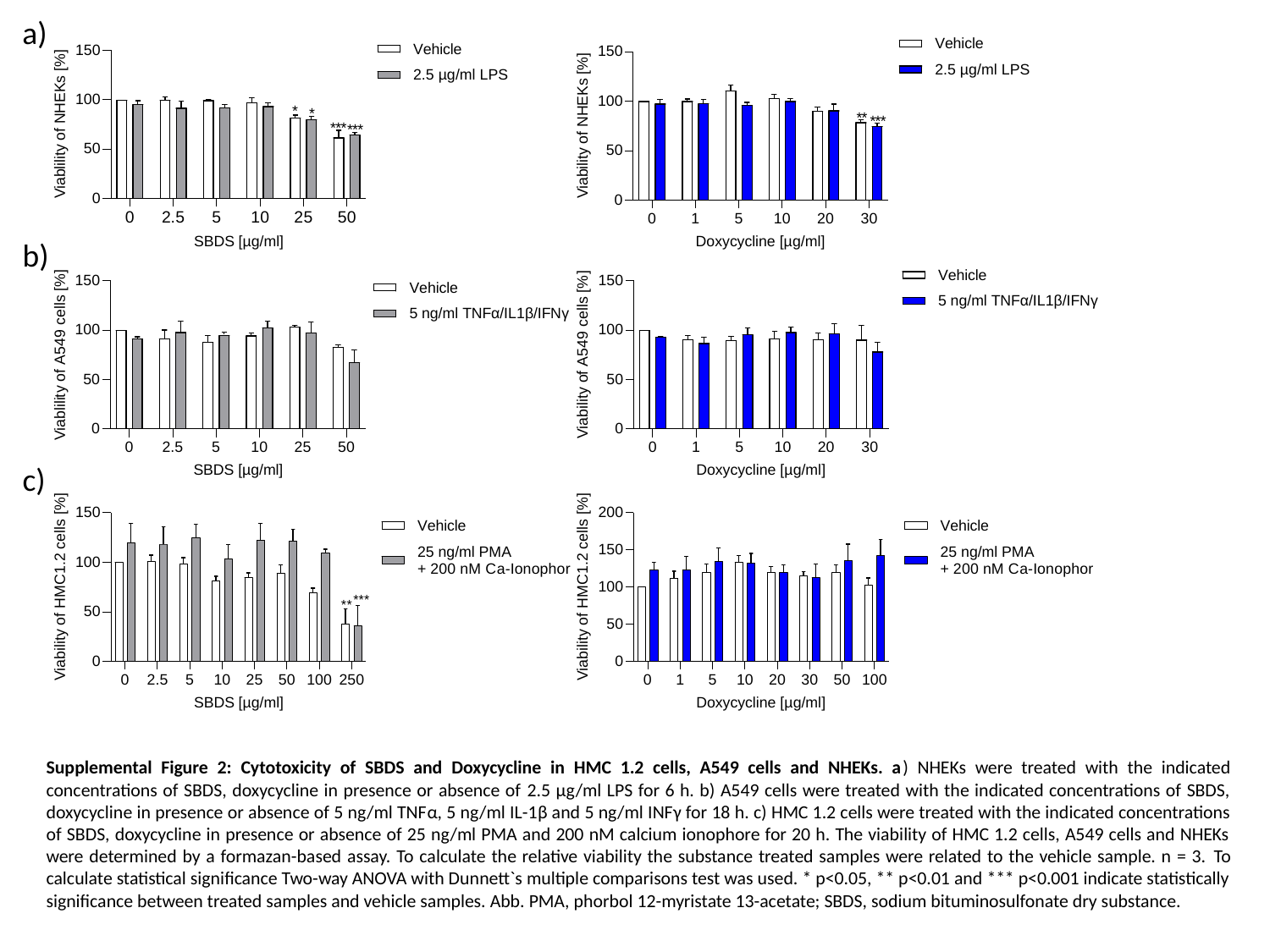

a)
b)
c)
Supplemental Figure 2: Cytotoxicity of SBDS and Doxycycline in HMC 1.2 cells, A549 cells and NHEKs. a) NHEKs were treated with the indicated concentrations of SBDS, doxycycline in presence or absence of 2.5 μg/ml LPS for 6 h. b) A549 cells were treated with the indicated concentrations of SBDS, doxycycline in presence or absence of 5 ng/ml TNFα, 5 ng/ml IL-1β and 5 ng/ml INFγ for 18 h. c) HMC 1.2 cells were treated with the indicated concentrations of SBDS, doxycycline in presence or absence of 25 ng/ml PMA and 200 nM calcium ionophore for 20 h. The viability of HMC 1.2 cells, A549 cells and NHEKs were determined by a formazan-based assay. To calculate the relative viability the substance treated samples were related to the vehicle sample. n = 3. To calculate statistical significance Two-way ANOVA with Dunnett`s multiple comparisons test was used. * p<0.05, ** p<0.01 and *** p<0.001 indicate statistically significance between treated samples and vehicle samples. Abb. PMA, phorbol 12-myristate 13-acetate; SBDS, sodium bituminosulfonate dry substance.

## Slide 3
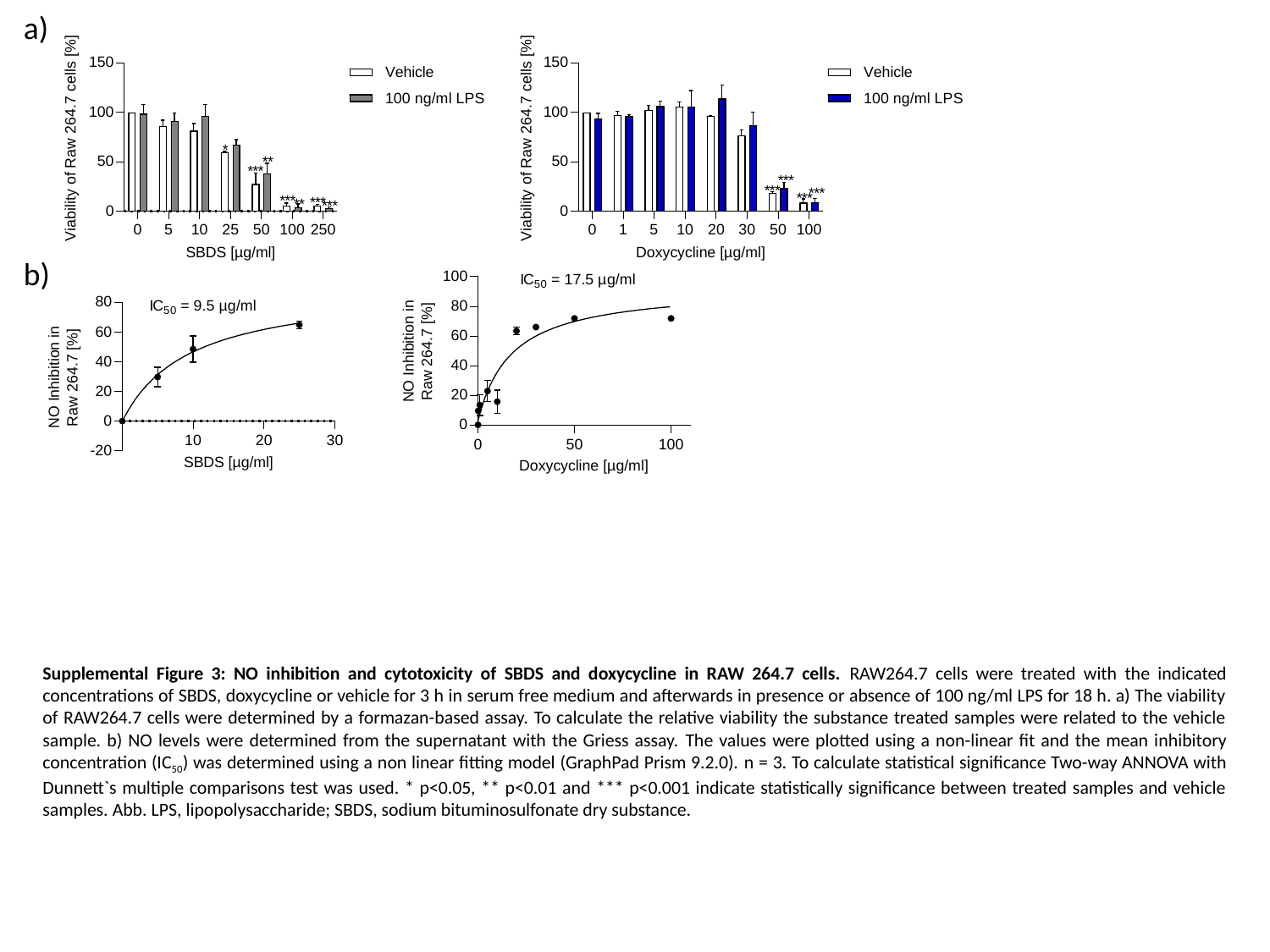

a)
b)
Supplemental Figure 3: NO inhibition and cytotoxicity of SBDS and doxycycline in RAW 264.7 cells. RAW264.7 cells were treated with the indicated concentrations of SBDS, doxycycline or vehicle for 3 h in serum free medium and afterwards in presence or absence of 100 ng/ml LPS for 18 h. a) The viability of RAW264.7 cells were determined by a formazan-based assay. To calculate the relative viability the substance treated samples were related to the vehicle sample. b) NO levels were determined from the supernatant with the Griess assay. The values ​​were plotted using a non-linear fit and the mean inhibitory concentration (IC50) was determined using a non linear fitting model (GraphPad Prism 9.2.0). n = 3. To calculate statistical significance Two-way ANNOVA with Dunnett`s multiple comparisons test was used. * p<0.05, ** p<0.01 and *** p<0.001 indicate statistically significance between treated samples and vehicle samples. Abb. LPS, lipopolysaccharide; SBDS, sodium bituminosulfonate dry substance.

## Slide 4
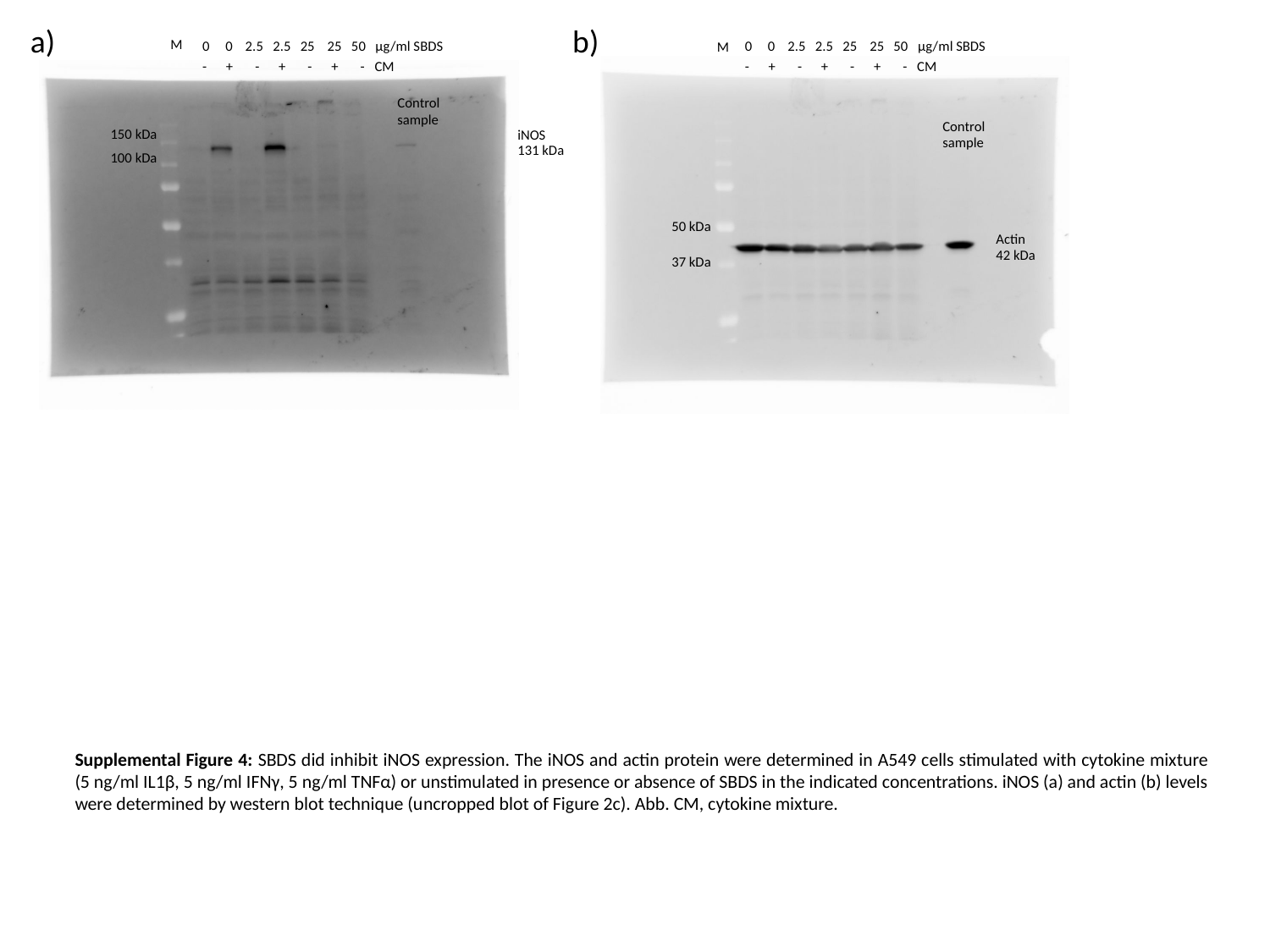

a)
b)
M
0 0 2.5 2.5 25 25 50 μg/ml SBDS
0 0 2.5 2.5 25 25 50 μg/ml SBDS
M
- + - + - + - CM
- + - + - + - CM
Control sample
Control sample
150 kDa
iNOS
131 kDa
100 kDa
50 kDa
Actin
42 kDa
37 kDa
Supplemental Figure 4: SBDS did inhibit iNOS expression. The iNOS and actin protein were determined in A549 cells stimulated with cytokine mixture (5 ng/ml IL1β, 5 ng/ml IFNγ, 5 ng/ml TNFα) or unstimulated in presence or absence of SBDS in the indicated concentrations. iNOS (a) and actin (b) levels were determined by western blot technique (uncropped blot of Figure 2c). Abb. CM, cytokine mixture.

## Slide 5
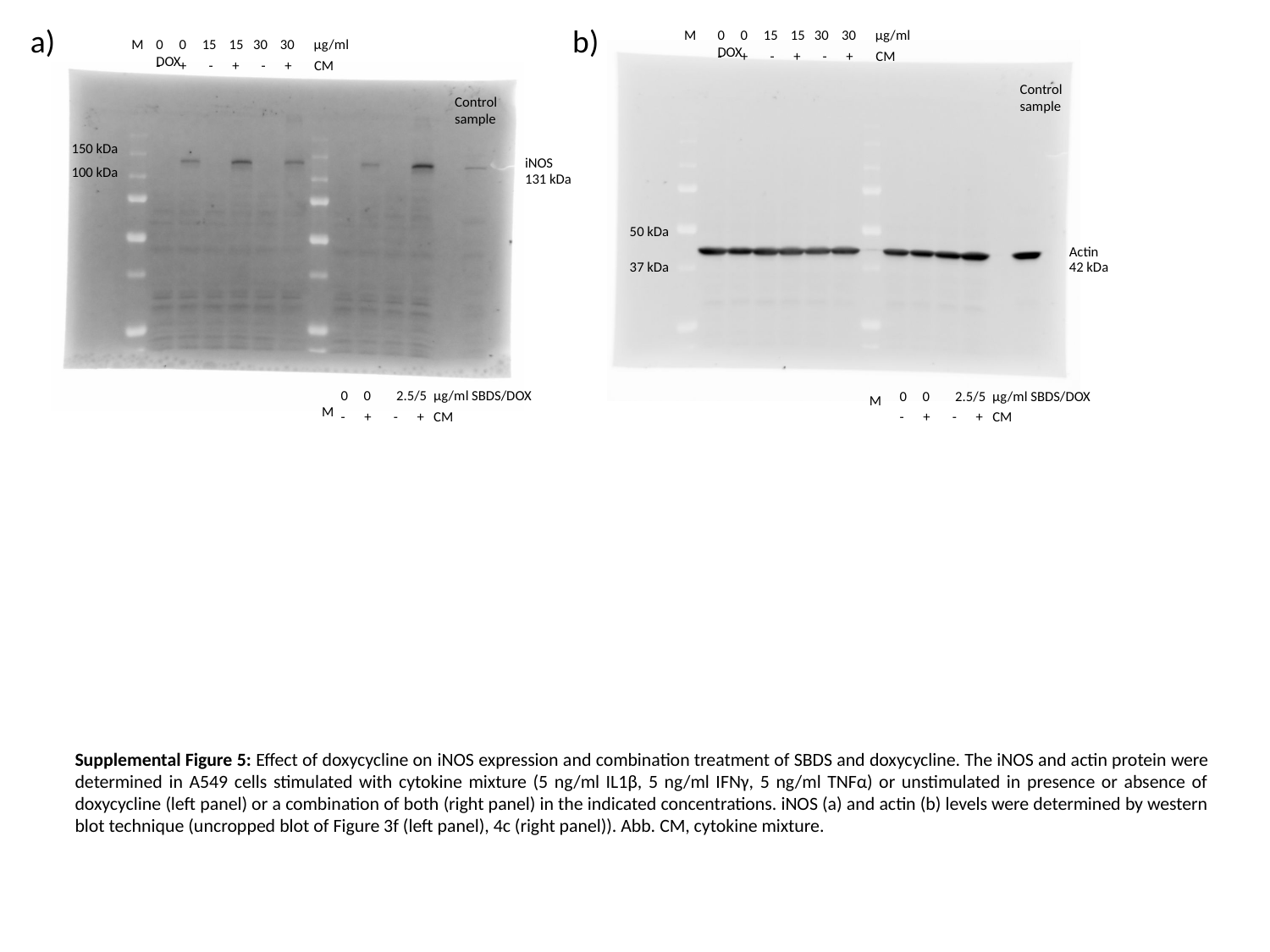

a)
b)
M
0 0 15 15 30 30 μg/ml DOX
M
0 0 15 15 30 30 μg/ml DOX
- + - + - + CM
- + - + - + CM
Control sample
Control sample
150 kDa
iNOS
131 kDa
100 kDa
50 kDa
Actin
42 kDa
37 kDa
0 0 2.5/5 μg/ml SBDS/DOX
0 0 2.5/5 μg/ml SBDS/DOX
M
M
- + - + CM
- + - + CM
Supplemental Figure 5: Effect of doxycycline on iNOS expression and combination treatment of SBDS and doxycycline. The iNOS and actin protein were determined in A549 cells stimulated with cytokine mixture (5 ng/ml IL1β, 5 ng/ml IFNγ, 5 ng/ml TNFα) or unstimulated in presence or absence of doxycycline (left panel) or a combination of both (right panel) in the indicated concentrations. iNOS (a) and actin (b) levels were determined by western blot technique (uncropped blot of Figure 3f (left panel), 4c (right panel)). Abb. CM, cytokine mixture.
